# Non-thyroidal illness syndrome and cardiopulmonary bypass in children with congenital heart disease

**Published:** 2014

**Authors:** Kazem Babazadeh, Avisa Tabib, Peyman Eshraghi, Hooman Bakhshandeh, Hassan Zamani

**Affiliations:** 1 Non-Communicable Pediatric Diseases Research Center, Babol University of Medical Sciences, Babol, Iran.; 2 Heart Valve Disease Research Center, Rajaie Cardiovascular Medical Research Center, Iran University of Medical Sciences, Tehran, Iran.; 3 Mashhad University of Medical Sciences, Mashhad, Iran.

**Keywords:** Non-thyroidal Illness Syndrome, Cardiopulmonary bypass, Cyanosis, Inotropic drugs

## Abstract

***Background: ***The thyroid hormones influence on all metabolic pathways. After heart surgery using cardiopulmonary bypass (CPB), serum T3 decreases and remains low for at least 24 hours. Several studies on pediatric have reported reduction of thyroid hormones after heart surgery. This study aimed to investigate the status of thyroid function tests in children with CPB surgery.

***Methods:*** This study was carried out based on the available data on 132 children aged less than 15 years suffering from CHD. The patients underwent open heart surgery in Rajaie Center in Iran from January to November 2010. The thyroid hormone levels were measured shortly after admission, and postoperatively in Intensive Care Unit (ICU) and thereafter at 12, 24 and 48-hour intervals. The patients’ gender, age, weight, body mass index, heart disease details, previous cardiac surgeries, and cardiac surgery-related data such as pump time, aortic clamping time, hypothermia duration, postoperative hemodynamic status and postoperative use of inotropic drugs were recorded and analyzed

***Results:*** All patients showed a decrease in T3, T4 and TSH and an increase in T3-resin uptake after surgery. Eventually, 3 (3.2%) patients died. Preoperatively, there was a significant association between the reduction in the thyroid hormone levels and inotropic drugs as well as the type of the heart disease (p<0.05).

***Conclusion: ***There is an association between post-operative inotropic drugs administration and reduction thyroid hormones levels in patients undergoing congenital heart disease cardiopulmonary bypass surgery.

The cardiovascular impacts of the thyroid hormones may include increased contractility and cardiac output and reduced vascular resistance, all of which occur through several mechanisms.(1-3) In patients undergoing cardiac surgery using cardiopulmonary bypass (CPB), the non-thyroidal illness (NTI) syndrome can occur in the postoperative period due to stress (4). The mechanism of transient hypothyroidism following CPB is identified by hemodilution, endogenous factors such as glucocorticoids, tumor necrosis factor, and interlukin-6, or exogenous factors like dopamine administration or iodine skin preparation (4).

In the postoperative period, with the deterioration of the patient’s condition, the hypothalamic-pituitary-thyroid axis is suppressed, which in turn leads to a reduction in serum free T_4_ (5). 

In addition, because of the inhibition of type 1 monodeiodinase activity in non-thyroidal tissues, the production of T_3_ from T_4 _is diminished. As a result of this reduction, the mortality rate will increase (6). Low cardiac output syndrome and heart failure due to postoperative hypothyroidism occur at the same time as T3 is suppressed, especially within the first 48 postoperative hours (7, 8). The administration of T_3 _at this stage is quite likely to improve heart failure and increase cardiac output (9). We aimed to investigate whether patients with CPB develop postoperative NTI syndrome and determine the factors involved in the changes in the levels of thyroid hormone levels in the postoperative period.

## Methods

One hundred thirty-five patients aged between one month and 15 years (average age = 3.89±3.34 years) with congenital heart disease undergoing cardiac surgery (surgeries using CPB such as total correction of tetralogy of fallot, ventricular septal defect closure, Rastelli operation, aortic valve replacement, pulmonic valve replacement, and Senning and arterial switch for the transposition of great arteries)were signed up for our study at the Department of Pediatric Cardiology at Rajaie Cardiovascular, Medical and Research Center in Tehran, Iran, from January 2010 to November 2010. The study protocol was approved by the Institutional Ethics Committee.

To assess the levels of thyroid hormone, TSH, thyroxin, triiodothyronine, and T_3 _resin uptake (T_3_ru) were measured through different techniques. The patients’ blood samples were collected at regular intervals; i.e. on admission, immediately postoperatively, and at 12, 24, and 48 hours postoperatively. Then, the serum was separated from the blood by centrifusion and stored at 2-7 ºC until assay. The serum concentration of the hormones was measured via the Enzyme-Linked Immunosorbent Assay (ELISA) using Monobind Company kits. Additionally, T_3_ and T_4_ were measured using a competitive enzyme immunoassay (Type 5) and TSH was measured using immunoenzymometric assay (Type 3). Other data such as gender, age, weight, body mass index (BMI), previous cardiac surgeries, cardiac surgery-related information (e.g.pump time, aortic clamping time, and hypothermia duration), postoperative hemodynamic status in the intensive care unit (ICU), intubation period in the ICU, and the postoperative use of inotropic drugs were obtained from the patients' files. The exclusion criteria comprised the disease of other organs (e.g. liver, kidney, and central nervous system), endocrine abnormalities prior to hospitalization, and the use of any drugs that could impact the status of the thyroid hormones such as glucocorticoids, phenytoin, growth hormones, gonadal steroids, propiltiouracil, and Levothyroxine. 

Data were expressed as mean±SD for interval and count (%) for categorical variables. One Kolmogorov-Smirnov test sample was used to investigate the fitness of interval variables to normal distribution. Student's t test was applied to show the differences of the hormone levels between sub-groups of patient's characteristics. The correlations between serum levels of hormones and interval characteristics were determined using Pearson's correlation coefficients. Repeated measure analysis of variance (ANOVA) models were applied to investigate the trend of hormonal changes through the time. A p-value <0.05 was considered as statistically significant. SPSS 15 for Windows (SPSS Inc., Chicago, Illinois) was used for statistical analysis.

The data were analyzed via statistical tests such as student *t-*test, Kruskal-Wallis test, one-way ANOVA, and repeated measures ANOVA. The Pearson’s correlation coefficient (r) was used to demonstrate the correlations between the data, and multiple linear regression models were employed for the assessment of the modified relevance between the changes in the levels of thyroid hormone and the other variables discussed above.

## Results

In this study, 135 patients, including 89 (65.9%) males and 46 (34.1%) females on an average age of 3.89±3.34 years, undergoing cardiac surgery were studied. Their congenital heart defects included tetralogy of fallot, ventricular septal defect, atrial septal defect, pulmonary stenosis, aortic stenosis, transposition of great arteries, and single ventricle. Three patients died postoperatively and were removed from the study. Out of the 132 patients, 66 were cyanotic and the rest were non-cyanotic. Also, the previous palliative procedures or temporary cardiac surgeries were performed on 32 (24.2%) patients. The patients’ background and demographic characteristics are depicted in table 1.

**Table 1:** Demographic and general information of the study participants (n =132)

	**Descriptive Index**
Sex (F/M)	45/87
Age (years)	4±3.3
Congenital heart defects	
Cyanotic	66 (50%)
Non-cyanotic	66 (50%)
Previous surgery	32 (24%)
Body Mass Index(BMI)	15.03±2.67
Pump time(min)	92±44.82
Aortic clamping time (min)	55.14±32.41
Hypothermia time (min)	50.18±32.24
ICU stay (days)	3.96±3.89
Intubation period (hrs.)	34.21±49.98
Drugs	
Dopamine	34 (25.8%)
Epinephrine	33 (25%)
Milrinone	28 (21.2%)
Left ventricular ejection fraction (%)	64.68±12.12
Mortality	3 (2.3%)

**Figure 1:**
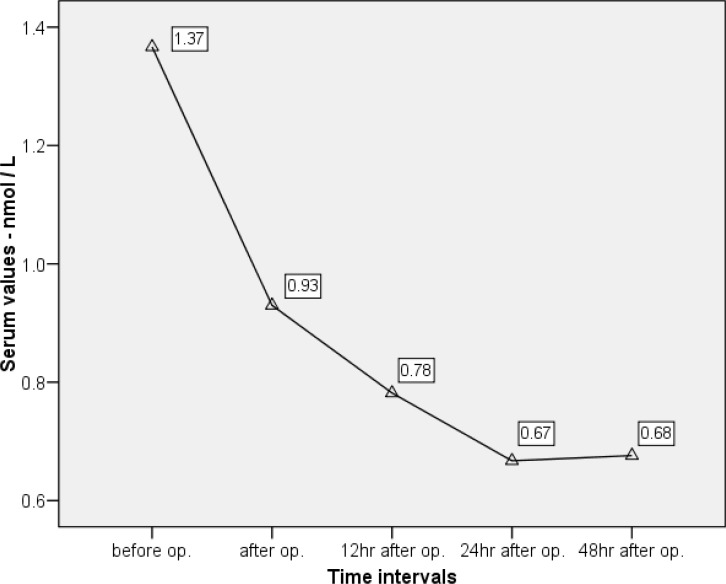
Diagram of changes in serum T_3_ based on sampling times

**Figure 2:**
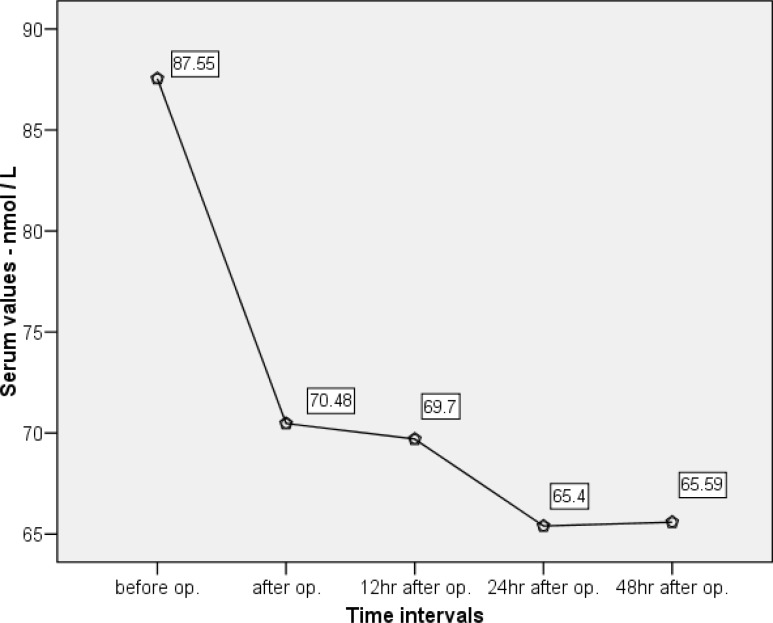
Diagram of changes in serum T_4_ based on sampling times

**Figure 3:**
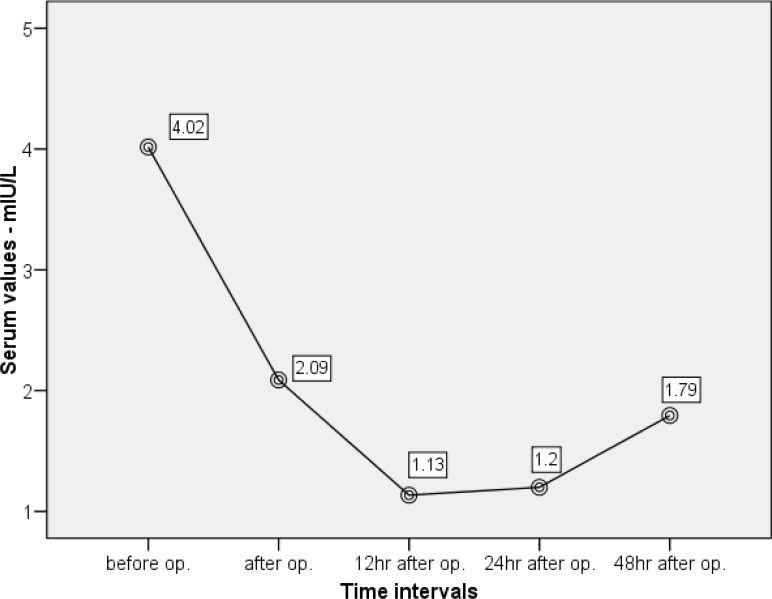
Diagram of changes in serum TSH based on sampling times

The status of hormone levels at different time intervals are summarized in table 2 and figures 1-3. All these patients demonstrated a decrease in serum TSH, T_4_, and T_3_ and an increase in T_3_RU after CPB. Repeated measures ANOVA revealed that the reductions in TSH, T_3_, and T_4_ were significant (p<0.001). 

According to the Bonferroni post-hoc test, there was a significant association between the average levels of T_3 _preoperatively, immediately postoperatively, and at 12 and 24 hours postoperatively (p<0.05). However, there was no evidence of such association between the T_3_ levels at 24 and 48 hours after surgery. In other words, T_3_ reached its plateau state at 24 hours postoperatively, and no further decline thereafter (figure 1).

There was an association between the average level of T_4 _preoperatively, immediately postoperatively, and one day postoperatively. Nevertheless, there was no such evidence immediately, at 12 hours, one day, and 2 days postoperatively. In other words, one day after surgery, the T_4_ level stayed the same without any changes (figure 2). As illustrated in figure 3, there was a sudden increase in T_3_RU until immediately after the operation, followed by a decline in the serum level lower than its normal preoperative level. 

The next diagram shows that the TSH level continued to drop until the 12^th^ postoperative hour, thereupon, it remained unchanged for the following 12 hours and then started to rise (figure 3). This means that probably the increase in TSH is preceded by a rise in T_3_ and T_4_.

The percentages of the decline in the TSH, T_3_, and T_4 _levels immediately postoperatively and in the ICU admission in comparison to their preoperative levels were 0-95.64%, 0-85%, and 0-54.78%, respectively. On the second postoperative day, these percentages in relation to their preoperative values were 0-98.85%, 0-90%, and 0-74.36%, respectively. The percentages of the decline of T_3_RU were 0-52.17% and 0-44.44% within the same time frame discussed above. The associations between the patients' baseline values in the levels of thyroid hormone and the patients’ characteristics were also investigated. The results are shown in table 3. Based on our statistical analyses, there was significant association between baseline T3 and T4 levels and gender, congenital heart disease, dopamine, epinephrine and milrinone (all *p-values *<0.05). 

Pearson’s correlation coefficient (r) revealed that there was significant linear correlation between the levels of thyroid hormone and some interval covariate (such as pump time, aortic clamping time, aortic clamping time, hypothermia time, ICU stay, intubation period, left ventricular ejection fraction and body mass index), however, all of them were not strong (all the "r" s were <0.5). The most important relations were observed in ICU stay (r= 0.48) and intubation period (r=0.49). 

**Table 2:** Comparison of serum thyroid hormone levels between different time intervals

	**Before op.**	**Immediately after op.**	**12hr after op.**	**24hr after op.**	**48hr after op.**	**P-value**
T3	1.36±0.42	0.92±0.34	0.78±0.32	0.66±0.29	0.67±0.27	<0.001
T4	87.54±17.74	70.47±16.43	69.70±17.37	65.40±17.92	65.59±20.56	<0.001
TSH	4.01±1.94	2.08±1.45	1.13±0.89	1.19±1.18	1.79±1.62	<0.001
T3ru	28.34±2.64	32.28±2.70	30.88±2.75	30.59±2.66	30.24±2.67	<0.001

**Table 3:** Associations between serum thyroid hormone levels at the baseline and background characteristics of the study participants

		**T3**		**T4**		**TSH**		**T3ru**
**Sex**								
Female (n=45)		0.66±0.28		66.14±20.93		1.67±1.37		30.08±2.57
Male (n=87)		0.70±0.26		64.51±20.02		2.01±2.01		30.55±2.85
p-value		0.42		0.66		0.23		0.33
**Congenital heart defects**								
Cyanotic (n=66)		0.62±0.25		58.71±19.73		1.69±1.74		31.21±2.44
Non-cyanotic (n=66)		0.73±0.28		72.46±19.16		1.89±1.49		29.98±2.75
p-value		0.02		<0.001		0.488		0.008
**Dopamine**								
Yes (n=34)		0.53±0.22		49.35±15.57		0.81±0.94		30.50±2.71
No (n=98)		0.72±0.27		71.22±19.08		2.13±1.67		30.15±2.66
p-value		<0.001		<0.001		<0.001		0.51
**Epinephrine**								
Yes(n=33)		0.55±0.23		51.15±16.08		1.57±1.39		30.81±2.35
No (n=99)		0.71±0.27		70.40±19.68		1.86±1.69		30.05±2.75
p-value		0.003		<0.001		0.38		0.12
**Milrinone**								
Yes (n=28)		0.52±0.22		49.64±14.69		1.3±1.44		30.82±2.26
No(n=104)		0.71±0.27		69.88±19.84		1.92±0.16		30.08±2.75
p-value		0.001		<0.001		0.05		0.197

## Discussion

All our patients demonstrated a significant decline in serum TSH, T_4_, and T_3_ and an increase in T_3_RU following cardiac surgery due to the NTI syndrome. The decline in TSH, T_4_, and T_3 _was discovered through our evaluations during the first 24 postoperative hours. These reductions continued until the 48^th^ postoperative hour, thereupon the thyroid hormones which had reached their lowest levels, remained in the same level for another day. There was a significant increase in T_3_RU after surgery, followed by a minor decline nearing its normal level 48 hours postoperatively. The reduction in the thyroid hormones might be due to the suppression of TSH release after CPB, which in turn was caused by the changes in the pituitary-thyroid gland axis.

In one study, TSH, triglyceride, and total T_4_, T_3_, and RT_3_ were measured in 14 patients pre-and postoperatively. The total levels of T_3_ and free T_3_ had a significant decrease in the first 24 hours after the bypass. In addition, the RT_3_ levels had an increase four times the normal level between 8 and 24 hours after the operation (10). In a different study on 14 children undergoing CPB, Murzi et al. observed a marked reduction in the total levels of T_3_, T_4_, free T_3_, and TSH as well as in the free T_3_ to free T_4_ ratio. In other words, each hormone reached its lowest level between 12 to 48 hours after CPB, thus indicating that the changes in the thyroid function and the conversion of T_4_ to T_3_ were triggered by CPB. (11) 

In two separate studies, T_3_, T_4_, free T_4_, and TSH were studied in children with congenital heart disease preoperatively throughout CPB, and up to 48 hours postoperatively. The results showed that T_3_ and TSH had a significant decrease, whereas free T_4 _had a postoperative rise (12, 13).

Holzer et al. reported that in children undergoing CPB, deiodinase activity diminished with a subsequent reduction in the conversion of T_4_ to T_3_ due to a decreasing concentration of plasma selenium (14). Allen et al. reported that in 12 children undergoing CPB, there was a significant reduction in the levels of thyroid hormone. In addition, there was a temporary association between the changes made in the metabolisms of these hormones and the severity of the patients’ conditions (15).

Plumpton and Haas discovered a significant association between the diminishing free T_3_ and TSH and the length of CPB. Also, they observed that infants who were on ventilation for at least the first 48 postoperative hours had an average free T_3_ of 0.9 pmol/L lower than those who were not (16). Barthowski reported a drop in the thyroid hormone levels in all their 20 infants with congenital heart disease who underwent cardiac surgery. These patients were divided into two groups: one group had prolonged recovery and the others did not. In the former group, the higher amounts of T_3_ in the ultra-filtrate and the lower amounts in the serum were detected (17).

However, there was no such association between the T_3_ levels and inotropic drug use. The bulk of serum T_3_ is produced in the peripheral tissues through the conversion of T_4_ to T_3_, and the rest is secreted by the thyroid gland (18). As a result, the NTI syndrome and inotropic drugs cannot influence the peripheral production of T_3_. Furthermore, the reason for a further decline in the thyroid hormones in patients who are on cardiotonic drugs can be the severity of the cardiac illness, which suppresses the TSH release post operation.

In the present study, cardiac illnesses were found to alter the thyroid hormone serum levels. As an illness deteriorates, changes in the levels of thyroid hormone become more significant. However, whether or not these drugs can result in further reductions in the thyroid hormone levels per se need further investigation.

Bettendorf et al. studied 132 children with congenital heart disease undergoing cardiac surgery and found that they had a low plasma concentration in TSH, T_3_, T_4_, immunoreactive T_4_ (IT_4_), and triglyceride, whereas their RT_3_ plasma concentration had an increase. In those patients whose T_3_ plasma concentration was less than 0.6 nmol/L, the mechanical ventilation was prolonged (19). But in our study, such result was not observed. Our results demonstrated that the T_4_ levels had a greater decline after the operation in the cyanotic patients than in the non-cyanotic patientsNonetheless, there was no evidence of such impact for TSH or T_3_.

In two separate studies, Kovacikova et al. compared the patients with delayed sternal closure with others with early sternal closure and found that the latter displayed dramatic thyroid suppression immediately after the operation, which did not return to its normal level until later (20-21). In another study, Brogan et al. measured the thyroid hormones before and after cardiac surgery and found that CPB had a far more significant impact on the thyroid hormone metabolism than the preoperative antiseptic (22). Unfortunately, we were not able to come to this conclusion. Assessing the impacts of hypothermia on the thyroid hormone alterations, Eggnum et al. found that hypothermia did not have any role in changing thyroxin in patients undergoing CPB (23).

In our study, there was no evidence suggesting that changes in the levels of thyroid hormone before the operation and other factors such as gender, weight, postoperative left ventricular ejection fraction, aortic clamping time, hypothermic time, intubation period in the ICU and previous cardiac surgeries were related. However, our results showed that different TSH, T_3_, and T_4_ levels were related to the BMI, pump time, and age of the patients, respectively. A decrease in the BMI and age and an increase in pump time were shown to be able to lead to a further decline in the TSH, T_3_, and T_4_ levels, respectively.

Finally, there was no evidence suggesting that the reduction in the levels of thyroid hormone after the operation and the type of congenital heart disease was correlated. However, the changes in the thyroid hormone levels in patients with single ventricle and transposition of great arteries were far more significant than those in other diseases. These changes were statistically not relevant.

In conclusion, it is clear that the normal function of the thyroid gland is vitally important to the proper function of the cardiovascular system. Acquired hypothyroidism in patients undergoing cardiac surgery can give rise to bradycardia, a decrease in cardiac output and heart contractility, and an increase in the systematic vascular resistance (4). Several studies have reported that whenever a congenital heart disease has complications, the cardiac operation proves far more difficult and there is a higher need for cardiotonic and inotropic drug administration pre-and postoperatively. As a result, changes in the serum thyroid hormones are more evident. The undesirable outcome of such changes could be treated by exogenous thyroid hormones.

The decline in the serum thyroid hormone levels in infants with congenital heart disease undergoing cardiac surgery depends on many factors such as inotropic drugs, age, operation time, and BMI. Therefore, it is recommended that the thyroid hormones be measured before and after the operation, especially in high-risk patients with congenital heart disease. These patients can be treated with Levothyroxine if the thyroid hormones are decreasing. In addition, it is recommended that the various data of the patients with the non-thyroidal Illness syndrome be studied for further treatment. These data include gender, pump time (long vs. short), disease type (cyanotic vs. non-cyanotic), and the use of cardiotonic drugs (pos. vs. neg.).

## Notes

**Funding:** Self-funded.
